# U‐Shaped Relationship Between White Blood Cell Counts and Incident Hypertension in Military Young Adults: The CHIEF Study, 2014–2020

**DOI:** 10.1002/iid3.70286

**Published:** 2025-10-15

**Authors:** Kun‐Zhe Tsai, Chia‐Wei Hong, Yun‐Chen Chang, Wei‐Chun Huang, Xuemei Sui, Carl J. Lavie, Gen‐Min Lin

**Affiliations:** ^1^ Department of Medicine Hualien Armed Forces General Hospital Hualien City Taiwan; ^2^ Department of Stomatology of Periodontology Mackay Memorial Hospital Taipei Taiwan; ^3^ Department of Periodontology, School of Dentistry National Defense Medical Center and Tri‐Service General Hospital Taipei Taiwan; ^4^ Department of Medicine, Tri‐Service General Hospital National Defense Medical Center Taipei Taiwan; ^5^ School of Nursing and Graduate Institute of Nursing China Medical University Taichung Taiwan; ^6^ Nursing Department China Medical University Hospital Taichung Taiwan; ^7^ College of Medicine National Yang Ming Chiao Tung University Taipei Taiwan; ^8^ Department of Critical Care Medicine Kaohsiung Veterans General Hospital Kaohsiung Taiwan; ^9^ Department of Exercise Science, Arnold School of Public Health University of South Carolina Columbia SC USA; ^10^ John Ochsner Heart and Vascular Institute, Ochsner Clinical School The University of Queensland School of Medicine New Orleans LA USA

**Keywords:** cohort study, hypertension, total leukocyte count, young adults

## Abstract

**Background:**

Systemic low‐grade inflammation, which is characterized by increased counts of blood leukocytes (white blood cells, WBCs), can lead to hypertension, while reduced WBC counts caused by the use of some substances, e.g., alcohol, can also lead to hypertension. However, there have been no studies on the association between WBC counts and the risk of hypertension in young adults.

**Methods:**

This cohort study conducted in Taiwan included a total of 2351 military personnel who were aged 18–39 years, were free of baseline hypertension and were followed up for incident hypertension from 2014 to the end of 2020. Resting blood pressure (BP) was measured during each annual health examination, while blood WBC counts and a biochemical panel were checked at baseline (2014). Incident hypertension was diagnosed as a systolic BP ≥ 130 mmHg and/or a diastolic BP ≥ 80 mmHg and/or the use of antihypertensive medications. Smooth curve fitting and multivariable Cox hazard proportional regression analysis, controlling for baseline sex, age, substance use, body mass index, BP, physical activity, serum uric acid level and kidney function, were used to determine the association between the baseline WBC count and incident hypertension.

**Results:**

During a median follow‐up of 6.1 years, 885 participants (37.6%) developed incident hypertension. A smoothing spline curve revealed a U‐shaped relationship between the WBC count and incident hypertension, with a turning point for the WBC count of 6.00 × 10^3^/μL. In the multivariable analysis, the WBC counts were classified into four quartiles, among which the 2ndquartile (5.56–6.40 × 10^3^/μL) was treated as the reference because it included the turning point. The 1st (lowest) WBC count quartile (4.03–5.55 × 10^3^/μL), the 3rd (high) WBC count quartile (6.41–7.43 × 10^3^/μL) and the 4th (highest) WBC count quartile (7.44–10.97 × 10^3^/μL) were associated with a higher risk of incident hypertension [hazard ratios and 95% confidence intervals: 1.50 (1.22, 1.83), 1.38 (1.14, 1.68) and 1.40 (1.16, 1.70), respectively]. The same pattern of association was observed across the sex, alcohol consumption, tobacco smoking, and physical activity subgroups.

**Conclusion:**

Among military personnel, not only increased WBC counts but also reduced WBC counts, within normal limits, were associated with a greater risk of incident hypertension. Those with low WBC counts may be vulnerable to infection, which subsequently leads to an increase in BP.

## 1 Introduction

Hypertension, a well‐established risk factor for cardiovascular diseases (CVDs), such as coronary heart disease and stroke, is traditionally considered to be regulated by physiological factors, including renal function, vasculature, and the sympathetic nervous system [1, 2]. Recent studies have demonstrated the pivotal role of immune blood cells, e.g., leukocytes (white blood cells, WBCs), in the development of hypertension [3, 4]. These findings revealed that WBCs could infiltrate vital organs, leading to organ dysfunction and high blood pressure (BP) [5, 6]. Moreover, in in vitro and in vivo animal studies, WBCs were closely involved in vascular remodeling, highlighting their potential to induce and perpetuate elevated BP levels [3, 7, 8].

WBC‐derived macrophages and other phagocytes may cause vascular injury, endothelial dysfunction, and the progression of arterial atherosclerotic diseases, leading to chronic low‐grade inflammation in the body [9, 10]. This inflammatory condition in turn plays a role in increasing microvascular capillary resistance, initiating platelet aggregation and increasing catecholamine levels, which can account for the development of hypertension [4]. However, research in this area remains limited, with the majority of data stemming from animal models.

Although a few human studies have revealed a potential association between elevated WBC counts and high BP levels, these studies predominantly focused on middle‐aged and older individuals [3, 4, 11, 12]. With sedentary lifestyles and overweight/obesity being prevalent in contemporary societies, a rising incidence of hypertension has been observed at younger ages [13, 14]. In contrast, low WBC counts within the normal reference range may be caused by the use of some specific substances, e.g., alcohol intake [15], and viral infection, which can also promote the development of hypertension [16]. It is important to understand the relationships of immune cells and representative low‐grade inflammation with hypertension in a population study [17, 18], particularly in young adults. Consequently, this study aimed to clarify the association between blood WBC counts and new‐onset hypertension in a population of military young adults while controlling for potential confounders.

## 2 Methods

### 2.1 Study Population

The Cardiorespiratory Fitness and Health in Eastern Armed Forces (CHIEF) [19] study is an observational military cohort study conducted in Eastern Taiwan [20]. In 2014, this study included 4,080 men and women aged 18 to 50 years from the Hualian Armed Forces General Hospital, the primary medical center serving military personnel in Eastern Taiwan. Baseline health and fitness assessments of participants were carried out between January 1 and December 31, 2014 [21, 22, 23]. Participants were excluded if they had baseline hypertension (*N* = 1024), were aged 40 years or older (*N* = 58), or had a baseline WBC count ≤ 4000/μL (*N* = 44) or ≥ 11,000/μL (*N* = 53). In addition, those who moved out of Eastern Taiwan military bases (*N* = 550) were excluded because they were lost to follow‐up. Consequently, 2351 participants were included in the final analysis. These participants did not have diabetes mellitus and were not using any antihypertensive or lipid‐lowering medications at the time of enrollment. The study protocol was reviewed and approved by the Institutional Review Board (IRB) of the Mennonite Christian Hospital in Hualien City, Taiwan (Approval No. 16‐05‐008). Written informed consent was obtained from all participants before their enrollment.

### 2.2 Baseline Health Assessment

BP measurements were acquired with participants in a seated position by using an oscillometric automatic device manufactured by Parama‐Tech Co. Ltd. If the initial systolic/diastolic BP surpassed 130/80 mmHg, a subsequent measurement was obtained following a 15‐min break, with the final BP value being the mean of the two readings [24, 25, 26]. Trained medical staff conducted anthropometric assessments, including waist circumference, body height, and body mass measurements, with participants in a standing position. Thereafter, the body mass index (BMI) was computed as the ratio of an individual′s body mass in kilograms to the square of their height in meters.

Each participant provided details about their substance use status, indicating whether they were active versus former or never users of alcohol, betel nut, and tobacco. Participants also reported their moderate‐intensity physical activity (PA) levels in the preceding 6‐month period, focusing on weekly leisure‐time run sessions, which were stratified into the following three categories: < 150, 150–299, and ≥ 300 min [27]. All the data were obtained through self‐report questionnaires.

Venous blood samples were procured from each study participant after an overnight fast for 12 h. Comprehensive laboratory investigations were conducted, including the quantification of serum concentrations of WBCs on an automated hematology analyzer (Sysmex XT‐2000i; Sysmex America, IL, USA) and biochemical testing of glucose, uric acid, blood urea nitrogen (BUN), and creatinine levels on an AU640 automated analyzer (Olympus, Kobe, Japan) [28]. The estimated glomerular filtration rate (eGFR) was derived according to the Modification of Diet in Renal Disease (MDRD) study equation to evaluate the renal functional capacity [29].

### 2.3 Definitions of Hypertension Phenotypes

In accordance with the guidelines set forth by the American College of Cardiology (ACC) and the American Heart Association (AHA) in 2017 [30], hypertension is defined as a systolic BP of 130 mmHg or higher and/or a diastolic BP of 80 mmHg or higher and/or the use of antihypertensive medication therapy. Isolated systolic hypertension (ISH) is defined as a systolic BP ≥ 130 mmHg with a diastolic BP < 80 mmHg, and isolated diastolic hypertension (IDH) is defined as a systolic BP < 130 mmHg with a diastolic BP ≥ 80 mmHg. Combined hypertension (CH) is defined as a systolic BP ≥ 130 mmHg and a diastolic BP ≥ 80 mmHg.

Furthermore, hypertension was defined according to the 7th report of the Joint National Committee (JNC 7) [31], in which the thresholds of systolic and diastolic BP for hypertension were raised to 140 and 90 mmHg, respectively.

### 2.4 Statistical Analysis

A smoothing spline curve was constructed to illustrate the associations between baseline total WBC counts and the risk of hypertension, with adjustments for age, sex, systolic and diastolic BP, BMI, substance use, PA levels, uric acid and BUN levels, and eGFR at baseline. The participants were divided into four groups according to the quartiles of the total WBC counts at baseline on the basis of our previous report on the association between total WBC counts and physical fitness [32] and a U‐shaped relationship between total WBC counts and the risk of hypertension by smooth curve fitting analysis in which the lowest risk was found within the 2nd low WBC count quartile [33]. Continuous variables are presented as the means ± standard deviations, and group differences were analyzed utilizing one‐way analysis of variance (ANOVA). Categorical data are reported as counts and percentages, and chi‐square tests were used for group comparisons.

The proportional hazards assumptions were confirmed by *p* values > 0.05. Cox proportional hazards regression models were used to examine the associations between WBC counts and the risk of hypertension by calculating hazard ratios (HRs) and 95% confidence intervals (CIs). The unadjusted model was set as Model 1. Multivariable Model 2 was adjusted for baseline age, sex, BMI, systolic BP and diastolic BP. Multivariable Model 3 was additionally adjusted for substance use and PA levels. Model 4 was further adjusted for uric acid level, BUN level, and eGFR. The associations between total WBC counts and specific hypertension types (ISH, IDH, and CH) were also explored by simultaneously adjusting for the covariates in Model 4. All the covariates were chosen on the basis of previously established associations [19].

Exploratory analyses were performed to examine potential effects on the association between the baseline WBC count and incident hypertension. This was achieved through stratified analyses and formal interaction tests according to sex, alcohol intake, tobacco use, and PA levels, with adjustments for the Model 4 covariates. Data analysis was performed by using free R version 4.3.2 (The R Foundation, http://www.R-project.org) and Empower‐Stats (http://www.empowerstats.com, X&Y Solutions Inc., Boston, MA). Statistical significance was defined as a two‐tailed *p* value < 0.05.

### 2.5 Results

**Figure 1:**
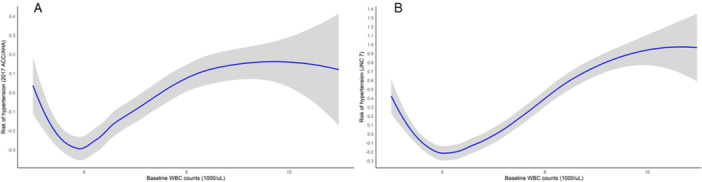
(A) and (B) display nonlinear, U‐shaped associations between baseline WBC counts and the risk of incident hypertension as per the 2017 ACC/AHA and JNC 7 guidelines, respectively. The turning points of the WBC counts were identified at 6,000/μL according to the 2017 ACC/AHA definition and 6,030/μL using the JNC 7 definition for hypertension.

The nonlinear, U‐shaped associations between the baseline WBC counts and the risk of incident hypertension according to the 2017 ACC/AHA and JNC 7 guidelines are shown in Figure 1A and 1B, respectively. The turning points of the total WBC counts were determined to be 6,000/μL on the basis of the 2017 ACC/AHA definition and 6,030/μL on the basis of the JNC 7 definition. Table 1 shows the baseline characteristics of the study population, which were categorized by the quartiles of the baseline total WBC counts as follows: 4.03–5.55 × 10³/μL, 5.56–6.40 × 10³/μL, 6.41–7.43 × 10³/μL, and 7.44–10.97 × 10³/μL. Significant differences were found among the groups in terms of systolic BP, diastolic BP, the BMI, waist circumference, alcohol intake, betel nut chewing, tobacco smoking, and uric acid and BUN levels, with a tendency to have greater levels in the higher total WBC count groups. However, there were no significant differences in baseline PA levels or fasting plasma glucose concentrations among the groups.

**Table 1:** Baseline characteristics of participants stratified by quartiles of total blood leukocyte counts.

	White blood cells counts				
	4.03–5.55 10^3^/uL (*N* = 589)	5.56–6.40 10^3^/uL (*N* = 588)	6.41–7.43 10^3^/uL (*N* = 591)	7.44–10.97 10^3^/uL (*N* = 583)	*p* value
Age, years	28.55 ± 5.73	28.01 ± 5.72	28.12 ± 5.66	27.74 ± 5.49	0.09
Male sex, %	514 (87.3)	521 (88.6)	526 (89.0)	512 (87.8)	0.79
SBP, mmHg	110.31 ± 10.82	112.42 ± 10.34	112.38 ± 10.20	113.10 ± 9.72	< 0.001
DBP, mmHg	65.25 ± 7.20	66.71 ± 6.90	66.51 ± 7.12	67.41 ± 6.99	< 0.001
BMI, kg/m^2^	23.72 ± 2.86	24.02 ± 3.04	24.50 ± 3.02	24.74 ± 3.25	< 0.001
Waist circumferences, cm	80.09 ± 8.02	80.77 ± 8.09	82.02 ± 7.98	82.98 ± 8.55	< 0.001
Substance use, %					
Alcohol drinking	206 (35.0)	219 (37.2)	245 (41.5)	258 (44.3)	0.005
Betel nut chewing	43 (7.3)	39 (6.6)	66 (11.2)	75 (12.9)	< 0.001
Tobacco smoking	168 (28.5)	172 (29.3)	233 (39.4)	261 (44.8)	< 0.001
PA levels, %					
< 150 min/week	123 (20.9)	136 (23.1)	146 (24.7)	128 (22.0)	0.24
150–299 min/week	229 (38.9)	226 (38.4)	231 (39.1)	203 (34.8)	
≥ 300 min/week	237 (40.2)	226 (38.4)	214 (36.2)	252 (43.2)	
Blood test					
WBC, 10^3^/uL	4.96 ± 0.40	5.99 ± 0.24	6.87 ± 0.29	8.54 ± 0.88	< 0.001
Fasting glucose, mg/dL	92.08 ± 10.01	92.75 ± 13.87	92.75 ± 9.42	93.33 ± 16.79	0.43
Uric acid, mg/dL	6.21 ± 1.29	6.42 ± 1.33	6.47 ± 1.41	6.62 ± 1.39	< 0.001
BUN, mg/dL	12.45 ± 2.79	12.48 ± 2.82	12.90 ± 2.90	12.58 ± 2.89	0.02
eGFR, mL/min/1.73m^2^	101.28 ± 14.11	101.15 ± 15.56	101.18 ± 14.05	101.67 ± 15.58	0.92

*Note:* Continuous variables are expressed as mean ± standard deviation (SD), and categorical variables as *N* (%)

Abbreviations: BMI, body mass index; BUN, blood urea nitrogen; CH, combined hypertension; DBP, diastolic blood pressure; eGFR, estimated glomerular filtration rate; IDH, isolated diastolic hypertension; ISH, isolated systolic hypertension; PA, physical activity; SBP, systolic blood pressure; WBC, white blood cells.

Over a median follow‐up period of 6.1 years, 885 participants (37.6%) developed hypertension according to the 2017 ACC/AHA guidelines, whereas 117 patients (5.0%) developed hypertension according to the JNC 7 criteria. Supplemental Table 1 outlines a two‐stage linear regression model, indicating a significant threshold effect between the baseline total WBC count and the risk of hypertension on the basis of the 2017 ACC/AHA and JNC 7 definitions. For both definitions, the association with hypertension followed a U‐shaped pattern. For the 2017 ACC/AHA definition, the inflection point (K) for the WBC counts was 6,000/μL. Below this threshold, the risk for developing new‐onset hypertension decreased significantly with a higher WBC count [HR: 0.79 (95% CI: 0.63–0.99)], whereas when the WBC count was above 6,000/μL, the risk significantly increased with a higher WBC count [HR: 1.12 (95% CI: 1.04–1.20)]. The log‐likelihood ratio yielded a *p* value < 0.001. With respect to the JNC 7 definition, the inflection point (K) for the total WBC counts was 6030/μL. When the WBC count was greater than this threshold, the risk for developing hypertension significantly increased with a higher WBC count [HR: 1.27 (95% CI: 1.06–1.53)], whereas there was no significant association for the risk of hypertension when the WBC count was lower than 6030/μL. The log‐likelihood ratio analysis also yielded *p* values < 0.001.

Table 2 shows the associations between baseline WBC counts, which were categorized by quartiles, and the risk of hypertension. Compared with the 2nd quartile (5560–6400/μL), which was treated as the reference, the risk of hypertension based on the 2017 ACC/AHA criteria was significantly greater for the 1st quartile (4030–5550/μL), the 3rd quartile (6410–7430/μL), and the 4th quartile (7440–10,970/μL) [HR: 1.75 (95% CI: 1.42–2.15); HR: 1.50 (95% CI: 1.23–1.83); and HR: 1.80 (95% CI: 1.48–2.19), respectively]. However, when the JNC 7 criteria were used, compared with the 2nd quartile, only the 4th quartile had a greater risk of incident hypertension [HR: 1.30 (95% CI: 1.02–1.65)] according to the unadjusted model, and there were no significant associations for the other quartiles. After adjustments for potential covariates were made, the total WBC counts were no longer associated with the risk of incident hypertension.

**Table 2:** Multivariable cox regression analysis for incident hypertension with baseline white blood cells counts.

			Model 1		Model 2		Model 3		Model 4	
	*N*	Events	HR (95% CI)	*p* value	HR (95% CI)	*p* value	HR (95% CI)	*p* value	HR (95% CI)	*p* value
2017 ACC/AHA criteria										
4.03–5.55 10^3^/uL	589	208	1.22 (1.00–1.49)	0.04	1.45 (1.19–1.77)	< 0.001	1.45 (1.19–1.77)	< 0.001	1.50 (1.22–1.83)	< 0.001
5.56–6.40 10^3^/uL	588	188	1.00 (Reference)		1.00 (Reference)		1.00 (Reference)		1.00 (Reference)	
6.41–7.43 10^3^/uL	591	235	1.37 (1.13–1.66)	0.001	1.37 (1.13–1.67)	0.001	1.38 (1.13–1.67)	0.001	1.38 (1.14–1.68)	0.001
7.44–10.97 10^3^/uL	583	254	1.49 (1.23–1.80)	< 0.001	1.40 (1.16–1.70)	< 0.001	1.42 (1.17–1.72)	< 0.001	1.40 (1.16–1.70)	0.001
JNC 7 criteria										
4.03–5.55 10^3^/uL	589	28	1.27 (0.73–2.19)	0.39	1.59 (0.91–2.76)	0.09	1.60 (0.92–2.77)	0.09	1.63 (0.94–2.84)	0.08
5.56–6.40 10^3^/uL	588	24	1.00 (Reference)		1.00 (Reference)		1.00 (Reference)		1.00 (Reference)	
6.41–7.43 10^3^/uL	591	26	1.18 (0.68–2.06)	0.54	1.17 (0.67–2.04)	0.57	1.15 (0.66–2.01)	0.61	1.12 (0.64–1.96)	0.68
7.44–10.97 10^3^/uL	583	39	1.77 (1.06–2.95)	0.02	1.60 (0.96–2.68)	0.068	1.55 (0.92–2.60)	0.09	1.50 (0.89–2.53)	0.12

*Note:* Data are presented as hazard ratio (HR) and 95% confidence interval (CI) using multivariable Cox regression analysis.

Abbreviations: ACC, American College of Cardiology; AHA, American Heart Association; JNC 7, the 7th report of the Joint National Committee.

Model 1 was crude model.

Model 2 adjusted for baseline age, sex, systolic blood pressure, diastolic blood pressure and body mass index.

Model 3 adjusted for variables in the Model 2 and baseline substances use and physical activity.

Model 4 adjusted for variables in the Model 3 and baseline serum uric acid, blood urea nitrogen and estimated glomerular filtration rate.

The results of multiple Cox regression analyses of the associations between baseline WBC count quartiles and incident hypertension phenotypes are shown in Table 3. With the 2017 ACC/AHA criteria, significant associations were found across various quartiles for specific hypertension phenotypes compared with the 2nd quartile, which was treated as the reference group. Regarding ISH, a higher risk was observed for the 1st quartile [HR: 1.72 (95% CI: 1.24–2.39)] and the 3rd quartile [HR: 1.57 (95% CI: 1.14–2.16)]. An increased risk for IDH was noted for the 1st quartile [HR: 1.64 (95% CI: 1.21–2.23)] and the 4th quartile [HR: 1.54 (95% CI: 1.15–2.07)]. For CH, a greater risk was found for the 3rd quartile [HR: 1.62 (95% CI: 1.06–2.46)] and the 4th quartile [HR: 1.72 (95% CI: 1.13–2.60)]. In contrast, when the JNC 7 criteria for hypertension were used, a significant association with ISH was observed only for the 4th quartile [HR: 3.07 (95% CI: 1.10–8.55)].

**Table 3:** Multiple cox regression analysis for incident hypertension by various phenotypes with baseline white blood cells counts.

			IDH			IDH			CH	
	*N*	Events	HR (95% CI)	*p* value	Events	HR (95% CI)	*p* value	Events	HR (95% CI)	*p* value
2017 ACC/AHA criteria										
4.03–5.55 10^3^/uL	589	82	1.72 (1.24–2.39)	0.001	90	1.64 (1.21–2.23)	0.001	36	1.52 (0.96–2.41)	0.07
5.56–6.40 10^3^/uL	588	70	1.00 (Reference)		79	1.00 (Reference)		39	1.00 (Reference)	
6.41–7.43 10^3^/uL	591	88	1.57 (1.14–2.16)	0.005	93	1.27 (0.94–1.73)	0.11	54	1.62 (1.06–2.46)	0.02
7.44–10.97 10^3^/uL	583	80	1.33 (0.95–1.84)	0.08	115	1.54 (1.15–2.07)	0.004	59	1.72 (1.13–2.60)	0.01
JNC 7 criteria										
4.03–5.55 10^3^/uL	589	4	1.27 (0.33–4.80)	0.72	16	1.39 (0.69–2.79)	0.35	8	4.66 (0.97–22.31)	0.054
5.56–6.40 10^3^/uL	588	5	1.00 (Reference)		17	1.00 (Reference)		2	1.00 (Reference)	
6.41–7.43 10^3^/uL	591	8	1.68 (0.54–5.22)	0.36	14	0.89 (0.43–1.81)	0.75	4	1.86 (0.33–10.26)	0.47
7.44–10.97 10^3^/uL	583	16	3.07 (1.10–8.55)	0.03	16	0.95 (0.47–1.91)	0.90	7	2.88 (0.59–14.09)	0.19

*Note:* Data are presented as hazard ratio (HR) and 95% confidence interval (CI) using multivariable Cox regression analysis with adjustments for baseline age, sex, systolic blood pressure, diastolic blood pressure, body mass index, substances use, physical activity, serum uric acid, blood urea nitrogen and estimated glomerular filtration rate.

Abbreviations: ACC, American College of Cardiology; AHA, American Heart Association; CH, combined hypertension; IDH, isolated diastolic hypertension; ISH, isolated systolic hypertension; JNC 7, the 7th report of the Joint National Committee.

The results of the subgroup analysis for the association between the baseline WBC count and incident hypertension on the basis of the 2017 ACC/AHA hypertension guidelines are shown in Table 4. There was no significant difference across the subgroups based on sex, alcohol consumption, tobacco smoking, and PA levels, and all *p* values for interactions were > 0.05.

**Table 4:** Subgroup Analyses for the Associations between White Blood Cells Counts and Incident Hypertension.

	4.03–5.55 10^3^/uL		6.41–7.43 10^3^/uL		7.44–10.97 10^3^/uL	
	HR (95% CI)	*p* value	HR (95% CI)	*p* value	HR (95% CI)	*p* value
Sex						
Men	1.49 (1.21–1.83)	< 0.001	1.39 (1.13–1.69)	0.001	1.40 (1.15–1.71)	0.001
Women	1.60 (0.56–4.55)	0.37	1.09 (0.40–2.96)	0.85	1.04 (0.39–2.79)	0.93
*P* for interaction		0.87		0.90		0.92
Alcohol drinking						
Active	1.33 (0.97–1.84)	0.07	1.41 (1.04–1.91)	0.02	1.32 (0.98–1.78)	0.064
Never/former	1.56 (1.21–2.02)	0.001	1.32 (1.02–1.70)	0.03	1.41 (1.09–1.83)	0.008
*P* for interaction		0.41		0.76		0.81
Tobacco smoking						
Active	1.77 (1.23–2.54)	0.002	1.79 (1.28–2.51)	0.001	1.50 (1.08–2.09)	0.01
Never/former	1.56 (1.21–2.02)	0.001	1.32 (1.02–1.70)	0.03	1.41 (1.09–1.83)	0.008
*P* for interaction		0.35		0.07		0.79
Physical Activity						
< 150 min/week	1.73 (1.10–2.71)	0.01	1.26 (0.83–1.92)	0.26	1.25 (0.81–1.94)	0.30
150–299 min/week	1.56 (1.13–2.16)	0.007	1.58 (1.15–2.17)	0.004	1.41 (1.02–1.94)	0.03
≥ 300 min/week	1.37 (1.01–1.88)	0.04	1.30 (0.95–1.78)	0.09	1.46 (1.09–1.97)	0.01
*P* for interaction		0.29		0.80		0.74

*Note:* Data are presented as hazard ratio (HR) and 95% confidence intervals (CI) using multivariable Cox regression analysis with adjustments for baseline age, sex, systolic blood pressure, diastolic blood pressure, body mass index, substances use, physical activity, serum uric acid, blood urea nitrogen and estimated glomerular filtration rate.

Total WBC Counts 5.56–6.40 10^3^/uL was treated as the reference group.

## 3 Discussion

This study revealed a U‐shaped relationship between baseline WBC counts and the risk of hypertension in young adults, according to the 2017 ACC/AHA or JNC 7 criteria. Our findings indicated that baseline WBC counts were negatively associated with the risk of hypertension when the WBC count fell within the low WBC count quartile of 4030–5550/μL and positively associated when the WBC count was within the upper two WBC count quartiles (6410–10,970/μL), with 6000/μL being the optimal WBC count associated with the lowest risk of hypertension. Furthermore, the association between the total WBC counts and incident hypertension was not affected by sex, alcohol consumption, tobacco smoking, or the PA level. Elevated WBC counts have been widely studied for their association with hypertension, often demonstrating a direct correlation between the total WBC count and the risk of developing hypertension. However, our study revealed a U‐shaped relationship between the total WBC counts and the risk of incident hypertension among young adults, which diverges from the linear relationship reported in previous research.

Two large cross‐sectional studies, which used Mendelian randomization analysis and a decision tree model, reported that a higher WBC count is correlated with an increased risk of hypertension [3, 11]. In addition, two cohort studies revealed that elevated WBC counts, particularly neutrophil counts [34], were a predictor of incident hypertension events, especially among women [4]. Furthermore, an elevated total WBC count is considered an independent predictor of CVD morbidity [35, 36]. Notably, a previous cohort study also revealed that elevated WBCs increased the risk of hypertension, even among individuals with WBC counts less than 6000/μL [12]. The mechanisms by which blood WBCs impact BP remain unclear. One hypothesis is that increased WBC counts are associated with arterial stiffness [37, 38], and this link might be mediated by reduced endothelial reactivity [39]. Another possibility is that higher WBC counts may lead to a greater sympathetic tone or directly increase peripheral vascular resistance by obstructing circulation in small blood vessels [40].

To the best of our knowledge, this is one of the first studies that revealed a U‐shaped relationship between total WBC counts and incident hypertension. This unique finding may be due to the fact that previous studies focused mainly on middle‐aged and older people, whereas our study specifically examined young adults, which may explain the observed difference in results. Existing studies have revealed that low WBC counts could be associated with the impact of, e.g., chronic alcohol intake, on nutritional status [41, 42], which may result in hypertension. Nutritional factors could explain the U‐shaped association between the total WBC count and hypertension risk observed in this study. In addition, viral infections, which are potentially related to low WBC counts, may serve as another mediating factor that increases BP levels [43, 44, 45], providing further insight into the U‐shaped relationship.

Further research on the role of total WBC counts in the pathogenesis of hypertension, particularly among young adults, is strongly recommended. This study might provide crucial insights into the early stage of hypertension and lead to novel immune system‐targeting treatments, which may be effective in younger adults. If a clear link is established, it could result in an affordable screening method, enabling early identification of at‐risk young adults. This study also revealed that baseline WBC counts were negatively associated with the risk of hypertension if the counts fell within the low WBC count quartile of 4030–5550/μL and positively associated when the counts were within the upper two WBC count quartiles (6410–10,970/μL), with 6000/μL being the optimal WBC count associated with the lowest risk of hypertension. With this inference, a new potential treatment approach for young adults with hypertension according to their baseline WBC counts should be investigated in the future.

### 3.1 Study Limitations

This study has notable strengths and limitations. A primary strength lies in the detailed baseline data, enabling adjustments for potential confounding variables, e.g., PA levels. In addition, the cohort of military personnel provides high internal validity, as they are predominantly young men (88.2%) with consistent healthcare access and consistent living conditions and are healthier relative to the general population. However, this demographic specificity, e.g., high PA levels among military personnel, may reduce the external validity of the study findings [46] and limit the applicability of the results to broader young adult populations. Furthermore, despite controlling for several potential covariates, unmeasured confounders, e.g., high‐sensitivity C‐reactive protein, which was not measured in this study [47], may influence outcomes. Additionally, the use of self‐report questionnaires for certain data introduces the potential for recall bias, which may affect the accuracy of the results. Finally, given the absence of information concerning recent nutritional status and infection status in this study, we speculated that malnutrition and/or viral infection might serve as mediating factors in the relationship between WBC counts and hypertension. Future studies should prospectively include other inflammatory biomarkers and collect information concerning nutritional status and infection history to validate the findings among more diverse young adult populations.

## 4 Conclusion

Our study demonstrated a novel U‐shaped relationship between baseline WBC counts and new‐onset hypertension in young adults. We found that the optimal WBC count associated with the lowest risk of incident hypertension was 6,000/μL. This study highlights that the total WBC count is an independent risk factor for new‐onset hypertension among young adults. Therefore, the total WBC count could serve as a potential hematological index for the early diagnosis of new‐onset hypertension in young adults.

## Author Contributions

**Kun‐Zhe Tsai:** formal analysis, software, writing – original draft. **Chia‐Wei Hong:** data curation, investigation, supervision, visualization, writing – review and editing. **Yun‐Chen Chang:** investigation, methodology, supervision, validation, visualization, writing – review and editing. **Wei‐Chun Huang:** conceptualization, investigation, supervision, validation, visualization, writing – review and editing. **Carl J. Lavie:** conceptualization, data curation, investigation, methodology, supervision, validation, visualization, writing – review and editing. **Gen‐Min Lin:** conceptualization, funding acquisition, investigation, methodology, project administration, resources, supervision, validation, visualization, writing – review and editing.

## Ethics Approval and Consent to Participate

The Institutional Review Board of the Mennonite Christian Hospital (No. 16‐05‐008) in Hualien, Taiwan approved access to the data for this study, and written informed consent was obtained from all participants.

## Conflicts of Interest

The authors declare no conflicts of interest.

## Supporting information


**Supplemental Table 1:** Threshold Effect Analysis of Baseline Total Leukocyte Counts on Incident Hypertension using Two‐Piecewise Cox Regression Model.

## Data Availability

The datasets generated and/or analyzed during the current study are not publicly available due to materials obtained from the military in Taiwan, which were confidential, but are available from the corresponding author on reasonable request.

## References

[1] A. Moiz , T. Zolotarova , and M. J. Eisenberg , “Outpatient Management of Essential Hypertension: A Review Based on the Latest Clinical Guidelines,” Annals of Medicine 56 (2024): 2338242, 10.1080/07853890.2024.2338242. 38604225 PMC11011233

[2] C. Iadecola , K. Yaffe , J. Biller , et al., “Impact of Hypertension on Cognitive Function: A Scientific Statement From the American Heart Association,” Hypertension 68 (2016): e67–e94, 10.1161/HYP.0000000000000053. 27977393 PMC5361411

[3] M. Siedlinski , E. Jozefczuk , X. Xu , et al., “White Blood Cells and Blood Pressure: A Mendelian Randomization Study,” Circulation 141 (2020): 1307–1317, 10.1161/CIRCULATIONAHA.119.045102. 32148083 PMC7176352

[4] A. Shankar , “Relationship Between White Blood Cell Count and Incident Hypertension,” American Journal of Hypertension 17 (2004): 233–239, 10.1016/j.amjhyper.2003.11.005. 15001197

[5] M. Hulsmans , H. B. Sager , J. D. Roh , et al., “Cardiac Macrophages Promote Diastolic Dysfunction,” Journal of Experimental Medicine 215 (2018): 423–440, 10.1084/jem.20171274. 29339450 PMC5789416

[6] T. J. Guzik , D. S. Skiba , R. M. Touyz , and D. G. Harrison , “The Role of Infiltrating Immune Cells in Dysfunctional Adipose Tissue,” Cardiovascular Research 113 (2017): 1009–1023, 10.1093/cvr/cvx108. 28838042 PMC5852626

[7] S. M. Krishnan , Y. H. Ling , B. M. Huuskes , et al., “Pharmacological Inhibition of the NLRP3 Inflammasome Reduces Blood Pressure, Renal Damage, and Dysfunction in Salt‐Sensitive Hypertension,” Cardiovascular Research 115 (2019): 776–787, 10.1093/cvr/cvy252. 30357309 PMC6432065

[8] J. M. Abais‐Battad , H. Lund , D. J. Fehrenbach , J. H. Dasinger , and D. L. Mattson , “Rag1‐Null Dahl SS Rats Reveal That Adaptive Immune Mechanisms Exacerbate High Protein‐Induced Hypertension and Renal Injury,” American Journal of Physiology‐Regulatory, Integrative and Comparative Physiology 315 (2018): R28–R35, 10.1152/ajpregu.00201.2017. 29537860 PMC6087888

[9] J. Sinisalo , J. Paronen , K. J. Mattila , et al., “Relation of Inflammation to Vascular Function in Patients With Coronary Heart Disease,” Atherosclerosis 149 (2000): 403–411, 10.1016/s0021-9150(99)00333-0. 10729391

[10] A. Mügge and J. A. Lopez , “Do Leukocytes Have a Role in Hypertension?,” Hypertension 17 (1991): 331–333, 10.1161/01.hyp.17.3.331. 1999364

[11] A. Mansoori , N. S. Farizani Gohari , L. Etemad , et al., “White Blood Cell and Platelet Distribution Widths Are Associated With Hypertension: Data Mining Approaches,” Hypertension Research 47 (2024): 515–528, 10.1038/s41440-023-01472-y. 37880498

[12] S. Ishida , S. Kondo , S. Funakoshi , et al., “White Blood Cell Count and Incidence of Hypertension in the General Japanese Population: ISSA‐CKD Study,” PLoS One 16 (2021): e0246304, 10.1371/journal.pone.0246304. 33529192 PMC7853436

[13] S. Abughazaleh , O. Obeidat , M. Tarawneh , Z. Qadadeh , and S. Alsakarneh , “Trends of Hypertensive Heart Disease Prevalence and Mortality in the United States Between the Period 1990‐2019, Global Burden of Disease Database,” Current Problems in Cardiology 49 (2024): 102621, 10.1016/j.cpcardiol.2024.102621. 38718934

[14] R. Del Pinto , C. Agabiti Rosei , A. Di Guardo , et al., “Prevalence, Clustering, and Current Management of Cardiovascular Risk Factors Upon First Referral to Hypertension Specialists: The APPROACH Study,” High Blood Pressure & Cardiovascular Prevention 31 (2024): 369–379, 10.1007/s40292-024-00650-4. 38780831 PMC11322322

[15] N. Nakanishi , H. Yoshida , M. Okamoto , Y. Matsuo , K. Suzuki , and K. Tatara , “Association of Alcohol Consumption With White Blood Cell Count: A Study of Japanese Male Office Workers,” Journal of Internal Medicine 253 (2003): 367–374. 12603505 10.1046/j.1365-2796.2003.01112.x

[16] M. Cecchini , T. Filippini , P. K. Whelton , et al., “Alcohol Intake and Risk of Hypertension: A Systematic Review and Dose‐Response Meta‐Analysis of Nonexperimental Cohort Studies,” Hypertension 81 (2024): 1701–1715, 10.1161/HYPERTENSIONAHA.124.22703. 38864208 PMC11251509

[17] M. Karakayali , T. Omar , I. Artac , İ. Rencuzogullari , Y. Karabag , and O. Demir , “The Relationship Between the Systemic Immune‐Inflammation Index and Reverse‐Dipper Circadian Pattern in Newly Diagnosed Hypertensive Patients,” Journal of Clinical Hypertension 25 (2023): 700–707, 10.1111/jch.14688. 37464585 PMC10423764

[18] M. Karakayalı , “Predictive Value of the SCORE, SCORE2, and Pooled Cohort Risk Equation Systems in Patients With Hypertension,” Turk Kardiyoloji Dernegi Arsivi‐Archives of the Turkish Society of Cardiology 51 (2023): 407–414, 10.5543/tkda.2023.74249. 37671521

[19] G. M. Lin , Y. H. Li , C. J. Lee , et al., “Rationale and Design of the Cardiorespiratory Fitness and Hospitalization Events in Armed Forces Study in Eastern Taiwan,” World Journal of Cardiology 8, no. 8 (2016): 464–471, 10.4330/wjc.v8.i8.464. 27621774 PMC4997527

[20] W. N. Liu , A. C. Feng , C. Y. Hsu , et al., “Mitral Valve Prolapse and Physical Performance in Asian Military Males: The CHIEF Heart Study,” Journal of Sports Sciences 41, no. 12 (2023): 1179–1186, 10.1080/02640414.2023.2260626. 37732628

[21] J. W. Lin , K. Z. Tsai , K. W. Chen , et al., “Sex‐Specific Association Between Serum Uric Acid and Elevated Alanine Aminotransferase in a Military Cohort: The CHIEF Study,” Endocrine, Metabolic & Immune Disorders ‐ Drug Targets 19, no. 3 (2019): 333–340, 10.2174/1871530319666181129163802. 30499423

[22] K. Z. Tsai , S. W. Lai , C. J. Hsieh , et al., “Association Between Mild Anemia and Physical Fitness in a Military Male Cohort: The CHIEF Study,” Scientific Reports 9, no. 1 (2019): 11165, 10.1038/s41598-019-47625-3. 31371766 PMC6671998

[23] F. Y. Su , S. H. Wang , H. H. Lu , and G. M. Lin , “Association of Tobacco Smoking With Physical Fitness of Military Males in Taiwan: The CHIEF Study,” Canadian Respiratory Journal 2020 (2020): 5968189, 10.1155/2020/5968189. 31998426 PMC6969999

[24] W. N. Liu , K. H. Lin , K. Z. Tsai , et al., “High Risk for Obstructive Sleep Apnea and Risk of Hypertension in Military Personnel: The CHIEF Sleep Study,” World Journal of Clinical Cases 11, no. 30 (2023): 7309–7317, 10.12998/wjcc.v11.i30.7309. 37969444 PMC10643064

[25] K. Z. Tsai , R. Y. Huang , W. C. Cheng , et al., “Association Between Dental Calculus and Hypertension Phenotypes in Highly Fit Adults: CHIEF Oral Health Study,” American Journal of Hypertension 36, no. 2 (2023): 102–108, 10.1093/ajh/hpac119. 36270011

[26] K. Z. Tsai , C. C. Chu , W. C. Huang , X. Sui , C. J. Lavie , and G. M. Lin , “Prediction of Various Insulin Resistance Indices for the Risk of Hypertension Among Military Young Adults: The CHIEF Cohort Study, 2014‐2020,” Cardiovascular Diabetology 23, no. 1 (2024): 141, 10.1186/s12933-024-02229-8. 38664804 PMC11046748

[27] G. M. Lin , K. Z. Tsai , Y. C. Chang , W. C. Huang , X. Sui , and C. J. Lavie , “Muscular Strength and Carotid Intima‐Media Thickness in Physically Fit Young Adults: The CHIEF Atherosclerosis Study,” Journal of Clinical Medicine 11, no. 18 (2022): 5462, 10.3390/jcm11185462. 36143108 PMC9501352

[28] W. N. Liu , Y. C. Hsu , Y. P. Lin , et al., “Substance Use and Incidence of Metabolic Syndrome Before Midlife Among Military Adults: The CHIEF Cohort Study,” Frontiers in Public Health 12 (2024): 1406524, 10.3389/fpubh.2024.1406524. 38894993 PMC11184061

[29] A. S. Levey , J. P. Bosch , J. B. Lewis , T. Greene , N. Rogers , and D. Roth , “A More Accurate Method to Estimate Glomerular Filtration Rate From Serum Creatinine: A New Prediction Equation,” Annals of Internal Medicine 130 (1999): 461–470, 10.7326/0003-4819-130-6-199903160-00002. 10075613

[30] L. D. Colantonio , J. N. Booth , A. P. Bress , et al., “2017 ACC/AHA Blood Pressure Treatment Guideline Recommendations and Cardiovascular Risk,” Journal of the American College of Cardiology 72 (2018): 1187–1197, 10.1016/j.jacc.2018.05.074. 30189994 PMC6346270

[31] A. V. Chobanian , “The Seventh Report of the Joint National Committee on Prevention, Detection, Evaluation, and Treatment of High Blood PressureThe JNC 7 Report,” Journal of the American Medical Association 289 (2003): 2560–2572, 10.1001/jama.289.19.2560. 12748199

[32] P. S. Chung , K. Z. Tsai , Y. P. Lin , Y. K. Lin , and G. M. Lin , “Association Between Leukocyte Counts and Physical Fitness in Male Military Members: The CHIEF Study,” Scientific Reports 10 (2020): 6082, 10.1038/s41598-020-63147-9. 32269281 PMC7142135

[33] H. Motulsky and A. Christopoulos , Fitting Models to Biological Data Using Linear and Nonlinear Regression: A Practical Guide to Curve Fitting (Oxford University Press, 2004), 12–47.

[34] Y. Tatsukawa , W. L. Hsu , M. Yamada , et al., “White Blood Cell Count, Especially Neutrophil Count, as a Predictor of Hypertension in a Japanese Population,” Hypertension Research 31 (2008): 1391–1397, 10.1291/hypres.31.1391. 18957810

[35] G. Schillaci , M. Pirro , G. Pucci , et al., “Prognostic Value of Elevated White Blood Cell Count in Hypertension,” American Journal of Hypertension 20 (2007): 364–369, 10.1016/j.amjhyper.2006.10.007. 17386341

[36] Y. Li , F. Fan , J. Jia , J. Li , Y. Huo , and Y. Zhang , “WBC Count Predicts the Risk of New‐Onset Peripheral Arterial Disease in a Chinese Community‐Based Population,” Hypertension Research 40 (2017): 932–936, 10.1038/hr.2017.64. 28490753

[37] M. Huang , F. Li , S. Chen , et al., “Total White Blood Cell Count Is Associated With Arterial Stiffness Among Hypertensive Patients,” Angiology 74 (2023): 657–663, 10.1177/00033197221115566. 35833809

[38] B. Jia , C. Jiang , Y. Song , et al., “Association Between White Blood Cell Counts and Brachial‐Ankle Pulse Wave Velocity in Chinese Hypertensive Adults: A Cross‐Sectional Study,” Angiology 73 (2022): 42–50, 10.1177/00033197211021199. 34164997

[39] M. S. V. Elkind , R. R. Sciacca , B. Boden‐Albala , et al., “Leukocyte Count Is Associated With Reduced Endothelial Reactivity,” Atherosclerosis 181 (2005): 329–338, 10.1016/j.atherosclerosis.2005.01.013. 16039287

[40] G. Friedman , “The Leukocyte Count: A Predictor of Hypertension,” Journal of Clinical Epidemiology 43 (1990): 907–911, 10.1016/0895-4356(90)90074-y. 2213079

[41] W. Y. Hsu , C. H. Wu , C. T. Hsieh , H. C. Lo , J. S. Lin , and M. D. Kao , “Low Body Weight Gain, Low White Blood Cell Count and High Serum Ferritin as Markers of Poor Nutrition and Increased Risk for Preterm Delivery,” Asia Pacific Journal of Clinical Nutrition 22 (2013): 90–99, 10.6133/apjcn.2013.22.1.05. 23353616

[42] M. D. Wirth , M. Sevoyan , L. Hofseth , N. Shivappa , T. G. Hurley , and J. R. Hébert , “The Dietary Inflammatory Index Is Associated With Elevated White Blood Cell Counts in the National Health and Nutrition Examination Survey,” Brain, Behavior, and Immunity 69 (2018): 296–303, 10.1016/j.bbi.2017.12.003. 29217263 PMC5857420

[43] R. R. Zhou , Y. H. Song , C. Y. Xu , et al., “Altered Counts and Mitochondrial Mass of Peripheral Blood Leucocytes in Patients With Chronic Hepatitis B Virus Infection,” Journal of Cellular and Molecular Medicine 28 (2024): e18440, 10.1111/jcmm.18440. 38890792 PMC11187856

[44] F. Yang and J. Luo , “The Association Between Hepatitis C Virus Infection Status and Blood Pressure in Adults in the United States: NHANES 1999‐2012,” Frontiers in Cellular and Infection Microbiology 14 (2024): 1401323, 10.3389/fcimb.2024.1401323. 38895738 PMC11183278

[45] P. Azami , R. G. Vafa , R. Heydarzadeh , et al., “Evaluation of Blood Pressure Variation in Recovered COVID‐19 Patients at One‐Year Follow‐up: A Retrospective Cohort Study,” BMC Cardiovascular Disorders 24 (2024): 240, 10.1186/s12872-024-03916-w. 38714940 PMC11075195

[46] D. A. Grimes and K. F. Schulz , “Bias and Causal Associations in Observational Research,” Lancet 359 (2002): 248–252, 10.1016/S0140-6736(02)07451-2. 11812579

[47] G. M. Lin , K. Liu , L. A. Colangelo , S. G. Lakoski , R. P. Tracy , and P. Greenland , “Low‐Density Lipoprotein Cholesterol Concentrations and Association of High‐Sensitivity C‐Reactive Protein Concentrations With Incident Coronary Heart Disease in the Multi‐Ethnic Study of Atherosclerosis,” American Journal of Epidemiology 183 (2016): 46–52, 10.1093/aje/kwv144. 26597828 PMC4690475

