# Supplementary material for: U‐Shaped Relationship Between White Blood Cell Counts and Incident Hypertension in Military Young Adults: The CHIEF Study, 2014–2020
Source: Immun Inflamm Dis. 2025 Oct 15;13(10):e70286. doi: 10.1002/iid3.70286 (PMC12521865; doi:10.1002/iid3.70286)
Supplement: Supplementary file 1 — Supplemental Table 1: Threshold Effect Analysis of Baseline Total Leukocyte Counts on Incident Hypertension using Two‐Piecewise Cox Regression Model. [file IID3-13-e70286-s001.docx]

**Supplemental Table 1.** Threshold Effect Analysis of Baseline Total Leukocyte Counts on Incident Hypertension using Two-Piecewise Cox Regression Model

|  |  | |  | Two-piecewise regression model | | | | | |
| --- | --- | --- | --- | --- | --- | --- | --- | --- | --- |
|  | Standard regression model | | Inflection point | <K-segment effect | |  | >K-segment effect | | Log likelihood ratio |
|  | HR (95% CI) | *P* value | (10^3^/uL) | HR (95% CI) | *P* value |  | HR (95% CI) | *P* value |  |
| 2017 ACC/AHA | 1.04 (0.99 – 1.09) | 0.08 | 6.00 | 0.79 (0.63 – 0.99) | 0.04 |  | 1.12 (1.04 – 1.20) | 0.002 | <0.001 |
| JNC 7 | 1.09 (0.96 – 1.24) | 0.17 | 6.03 | 0.68 (0.36 – 1.25) | 0.21 |  | 1.27 (1.06 – 1.53) | 0.009 | <0.001 |

Data are presented as hazard ratio (HR) and 95% confidence interval (CI) using multivariable Cox regression analysis with adjustments for baseline age, sex, systolic blood pressure, diastolic blood pressure, body mass index, substances use, physical activity, serum uric acid, blood urea nitrogen and estimated glomerular filtration rate.

Abbreviations: ACC, American College of Cardiology; AHA, American Heart Association; JNC 7, the 7th report of the Joint National Committee
